# Development and validation of a new TD-GC/MS method and its applicability in the search for human and animal decomposition products

**DOI:** 10.1007/s00216-014-7741-8

**Published:** 2014-03-16

**Authors:** E. Rosier, E. Cuypers, M. Dekens, R. Verplaetse, W. Develter, W. Van de Voorde, D. Maes, J. Tytgat

**Affiliations:** 1 Toxicology and Pharmacology, University of Leuven (KU Leuven), Campus Gasthuisberg, O&N2, Herestraat 49, PO Box 922, 3000 Leuven, Belgium; 2 CGSU/Disaster Victim Identification, Federal Police, Ruiterijlaan 2, 1040 Etterbeek, Belgium; 3 Imaging and Pathology Department, Division Forensic Biomedical Sciences, University of Leuven (KU Leuven), Campus Sint-Rafaël, Kapucijnenvoer 33, 3000 Leuven, Belgium

**Keywords:** TD-GC/MS, Human decomposition, Method development, Method validation

## Abstract

Differentiation between human and animal remains by means of analysis of volatile compounds released during decomposition is impossible since no volatile marker(s) specific for human decomposition has been established today. Hence, the identification of such a marker for human decomposition would represent great progression for the discovery of buried cadavers by analytical techniques. Cadaver dogs can be trained more efficiently, the understanding of forensic entomology can be enhanced, and the development of a portable detection device may be within reach. This study describes the development and validation of a new analytical method that can be applied in the search of such (a) specific marker(s). Sampling of the volatile compounds released by decomposing animal and human remains was performed both in a laboratory environment and outdoors by adsorption on sorbent tubes. Different coatings and several sampling parameters were investigated. Next, the volatile compounds were analyzed and identified by a thermal desorber combined with gas chromatography coupled to mass spectrometry (TD-GC/MS). Different GC columns were tested. Finally, the analytical method was validated using a standard mixture of nine representative compounds.

## Introduction

In forensic science, locating the body is one of the biggest challenges for police forces, but obviously of utmost importance for the progress of the investigation. Mostly, a multidisciplinary approach is used. This approach consists of the use of a variety of forensic techniques such as forensic ecology, forensic archeology, geophysical prospection, and several other means of forensic research. One of the investigative methods used is the use of the “dog human remains”. These dogs are trained mostly with nonspecific compounds such as butan-1,4-diamine and pentan-1,5-diamine by the Belgian Federal Police; there are only two trainers in Belgium in charge of these dogs. Frequently, trained cadaver dogs are used for the location of the buried bodies because of their olfactory capacity [1]. It may take days to find the body, while insects are attracted to the odor of decomposed bodies within minutes. Currently, it is a mystery which feature is responsible for their exceptional odor detection capacity. Depending on the decomposition stage, different insects are attracted [2]. In forensic entomology, the presence of the insects is being used to deduct the postmortem interval [3].

Several stages can be discriminated during decomposition of humans and animals [4–6]. The decomposition begins approximately 4 min after death. The first stage is the autolysis. The visible signs in this stage are fluid-filled blisters on the skin and skin slippage. Lipases, proteases, amylases, and other intracellular enzymes begin to dissolve the cells, which leads to the second stage, the putrefaction. This stage starts after approximately 48–72 h; soft tissue will decompose by the action of microorganisms (bacteria, fungi, and protozoa). A greenish discoloration of the skin appears. Gasses, liquids, and simple molecules are formed during this so-called bloating stage. When the outer layer of the skin breaks and the gasses escape, the active decay begins. Proteins, muscles, and fat break down. During the active decay, the liquefaction starts. This leads to skeletonization. Eventually, in the last stage or diagenesis, the bones degrade.

The decomposition process can be affected by many factors such as the characteristics of the body (e.g., age, body size, cause of death, clothing) or the location (e.g., temperature, moisture, type of soil, and, rodent and carnivore activity) [6].

A wide variety of volatile organic compounds (VOCs) are formed during the decomposition process. A few research groups have already studied these postmortem VOCs. They identified alkanes, alcohols, acids, esters, ketones, aldehydes, cyclic hydrocarbons, aromatic compounds, and sulphur- and nitrogen-containing compounds [7–16]. Different stages of the decomposition were studied using human cadavers or domestic pigs as human analog. As a result, different VOCs were linked to human decomposition, but only one group (Degreeff et al.) compared VOCs from human and animal remains. Only phenylethene and methyl benzoate were found to be more specific for human remains than for animal remains [15]. Cablk et al. analyzed the VOCs of animal tissue samples and compared their results to the published results for human samples. In comparison with Hoffman et al., they found 11 human specific compounds [16]. Literature evidence on human specific decomposition marker(s) is clearly limited.

In the previously described studies, several different analytical techniques were used. Because of the high volatility of VOCs and the wide spectrum of VOCs that are released during the decay process, a suitable sampling technique is essential in order to capture as many analytes as possible. A first sampling method that was used is thermal desorption (TD) where air is drawn through a sorbent tube and the VOCs can adsorb on the sorbent in the tube [8–12]. A second sampling technique is solid-phase micro-extraction (SPME). In this method, a fiber is immersed in the headspace and the molecules can adsorb in a passive way on the coating [14, 16]. There are also some rarely used techniques described such as scent transfer unit [15] or sorbent cartridges [13]. For detection and identification, gas chromatography coupled to mass spectrometry (GC/MS) was mostly used [8–12, 14–16]; one study used two-dimensional GC coupled to time-of-flight-MS (GCxGC-TOFMS) [13]. Unfortunately, the sensitivity of the published methods is not known and no validation was published to ensure the analytical quality.

In the first step of this study, a setup for human and animal remains was made for both, in a laboratory environment as well as outdoors. Next, the sampling of VOCs was optimized. The analysis and identification of the VOCs was done using TD-GC/MS. After optimizing the setup, sampling, and analysis parameters, the method of choice was validated. In the future, VOCs that are released during the decomposition of human and animal remains will be identified with this method and (a) human specific marker(s) (qualitatively or semiquantitatively) will be searched. The first outdoor experiments were performed in collaboration with the Disaster Victim Identification (DVI) of the Federal Police (Belgium), and further experiments will be performed to search (a) human specific marker(s).

Unlike the literature described above, where no validation data were published, the goal of our work was to develop a validated, highly specific and sensitive method for the detection of VOCs released during human and animal decomposition. Setup, sampling, and analysis steps were optimized accordingly.

## Materials and methods

### Chemicals and standards

The standard solution that was used to develop the method contains compounds, chosen based on the information found in the literature about VOCs involved in the decomposition of humans and animals. This solution is referred to as “development solution”. A mixture of alkanes, alcohols, ketones, aldehydes, acids, esters, aromatic compounds, and S- and N-containing compounds was dissolved in methanol. Pentane (>99.8 %), hexane (>99.7 %), heptane (>99.8 %), octane, (>99.7 %), nonane (>99.8 %), decane (>99.8 %), undecane (>99.8 %), ethyl propionate (>99 %), ethyl butyrate (>99 %), ethyl octanoate (>99 %), ethyl decanoate (>99 %), propyl acetate (>96 %), butanal (>99 %), pentanal (>97 %), hexanal (>97 %), heptanal (>95 %), octanal (>99 %), propanoic acid (>99.5 %), butanoic acid (>99 %), pentanoic acid (>99 %), octanoic acid (>99 %), dimethyl sulfide (>99 %), dimethyl disulfide (>98 %), methylpropyl disulfide (>90 %), 2-methylindole (>98 %), pentan-1,5-diamine (>97 %), butan-1,4-diamine (>99 %), benzene (>99.9 %), methylbenzene (>99.9 %), ethylbenzene (>99.5 %), 1,4-dimethylbenzene (>99.5 %), 1,3-dimethylbenzene (>99.5 %), 1,2-dimethylbenzene (>99.5 %), propylbenzene (>99.8 %), 2-ethyltoluene (>99 %) 4-ethyltoluene (>95 %), naphthalene (>99.7 %), and biphenyl (>99.8 %) were obtained from Sigma-Aldrich (Bornem, Belgium). Butanone (>99 %), pentan-2-one (>99 %), hexan-3-one (>98 %), heptan-2-one (>98 %), octan-2-one (>99 %), and triethylamine (>99 %) were obtained from Acros Organics (Geel, Belgium). Ethanol, propan-2-ol, butan-1-ol, decan-2-ol (98 %), ethyl acetate, and acetic acid were purchased from Merck (Darmstadt, Germany).

Validation was performed with a mixture of heptane, butan-1-ol, pentanal, pentan-2-one, ethyl propionate, dimethyl disulfide, butanoic acid, 1,2-dimethylbenzene, and octanal. A standard solution of 0.075 μL/mL (low concentration) and 0.75 μL/mL (high concentration) in methanol was prepared except for butan-1-ol (0.25 and 0.75 μL/mL) and butanoic acid (75 and 100 μL/mL). This solution is referred to as “validation solution”.

### Sampling

In order to compare different recipients, mice were decomposed in an erlenmeyer (2 L) (*n* = 4) and glass jars (1.062 L; Covera Packaging NV, Hoboken, Belgium). Different closures were tested for the jars (*n* = 2 for each closure): (1) fully closed with a cap, (2) closed with a small hole in the cap, (3) closed with parafilm, (4) closed during the decomposition except the first hour after sampling (in order to allow oxygen to reach the samples), and (5) completely open.

The VOC-containing air was sampled using an ACTI-VOC pump (Markes, Frankfurt, Germany). Air volumes with a range of 100 mL to 10 L combined with flow rates from 5 to 200 mL/min were tested.

### Method development

Six different coatings of the sorbent tubes were tested with the development solution. Carbosieve SIII, Carbotrap 202, and Carbopack B were obtained from Sigma-Aldrich (Bornem, Belgium); Tenax TA, Tenax/Unicarb, and Tenax/Carboxen 1003/Carbopack B were purchased from Camsco (Houston, TX).

As GC columns, a VF-5ms column (30 m × 0.25 mm × 0.25 μm) was tested first. Next, a VF-35ms column (30 m × 0.25 mm × 0.25 μm) and a VF-624ms column (60 m × 0.25 mm × 1.4 μm) from Agilent Technologies (Diegem, Belgium) were obtained and evaluated. The applied temperature gradient was also optimized.

### Description of method of choice

Before use, the sorbent tubes (prepacked Tenax TA tubes (200 mg, 89 mm × 6.4 mm o.d., Camsco, Houston, TX)) were conditioned for 1 h at 320 °C at a flow rate of 100 mL/min helium. One microliter of the validation solution was injected in a closed glass jar of 1.062 L with a septum-sealed hole in the cap (resulting in a concentration of 0.075 ppb (low) or 0.75 ppb (high), except for butan-1-ol and butanoic acid) and equilibrated for 1 h. With the ACTI-VOC pump, the air was drawn through the sorbent tubes for 20 min at 100 mL/min. After sampling, the sorbent tubes are closed with polytetrafluoroethylene (PTFE) analytical caps and placed into the TD autosampler for analysis.

The VOCs were analyzed using a TD-GC/MS (Turbomatrix 150 of Perkin Elmer (Zaventem, Belgium), 6890N-GC and 5975B-MS of Agilent Technologies (Diegem, Belgium)).

The TD was used for the desorption of the VOCs from the sorbent tubes. Primary desorption was accomplished by heating the sorbent tube to 300 °C for 30 min. A continuous helium gas flow (40 mL/min) transmitted the VOCs to a Tenax-coated cold trap held at 0 °C, where the VOCs are preconcentrated. In the second desorption stage, the trap was heated to 250 °C at a temperature rate of 99 °C/s and maintained at this temperature for 25 min; the flow of helium was going through the trap into the GC column via a transfer line at 250 °C. The VOCs were injected on the optimal GC column (VF-624ms, 60 m × 0.25 mm × 1.4 μm). The temperature of the GC oven was held at 40 °C for 1 min, increased to 80 °C at 1 °C/min, to 120 °C at 3 °C/min, and to 250 °C at 5 °C/min and maintained this latter temperature for 10 min. Helium (Air Products, Brussel, Belgium) carrier gas flow was going through the TD-GC/MS at a constant pressure (29 psi). The GC/MS interface was kept at a constant temperature of 280 °C. The electron impact ion source was used in positive mode at a temperature of 230 °C. The quadrupole mass analyzer was kept at 150 °C. Full scan spectra were recorded in a mass range of 15–400 amu.

### Method validation

#### Selectivity

To exclude any interference from contaminants, the air of a blank jar, closed for 1 h, was analyzed as described above. In every batch, a blank jar was also analyzed.

#### Recovery

The recovery was tested by comparing direct injection on the tube (1 μL of the validation solution was injected) and loading on the tube using the pump (1 μL of the validation solution was injected in a jar, equilibrated for 1 h and pumped on the tube) at two concentration levels (*n* = 2). The recovery is the ratio of the peak area of the injection using the pump over the peak area of the direct injection.

#### Stability

In order to test the stability, the validation solution was loaded on the tube like described above. During four consecutive days, two concentrations (low and high) were tested (*n* = 3 for each concentration). The tubes were closed with brass compression caps and stored at room temperature for a maximum of 4 days. The peak area of the analytes was plotted versus the time. Linear regression analysis was performed to check if the slope was significantly negative (*P* value < 0.05, which would indicate instability) with Graphpad Prism 6 (version 6.02, La Jolla, USA).

#### Linearity, limit of detection, and limit of quantification

The calibration concentrations were 0.075, 0.1, 0.18, 0.25, 0.5, and 0.75 μL/mL except for butan-1-ol (0.25, 0.3, 0.4, 0.5, 0.6, and 0.75 μL/mL) and butanoic acid (75, 80, 85, 90, 95, and 100 μL/mL) (*n* = 3 at each concentration). The peak area was plotted versus the concentration. The regression line was plotted with Graphpad Prism 6.

The limit of quantification (LOQ) was the lowest calibration point on the regression line, which had a signal-to-noise ratio (s/n) of minimum 10. The limit of detection (LOD) was based on a specific calibration curve in the range of the LOD (three lowest concentration points). It was calculated with the following formula:

$$ \mathrm{LOD}=3\times \left(\frac{\mathrm{S}{\mathrm{D}}_{\mathrm{intercept}}}{5}\right) $$

where SD_intercept_ is the standard deviation of the intercept, and *S* is the slope of the calibration curve.

#### Repeatability and intermediate precision

As described above, the validation solution (low and high concentration) was loaded on the sorbent tube. Repeatability (Rep) was tested by analyzing five tubes in 1 day. Intermediate precision (Int prec) was tested by analyzing eight tubes in 1 month. Repeatability and intermediate precision are expressed as percentage of relative standard deviation (%RSD) values.

#### Safe sampling volume

The safe sampling volume (SSV) is the volume that has to be drawn through the sorbent tube to start elution of the analyte off the sorbent tube. Two tubes were connected in series; the validation solution (high concentration) was loaded on the tubes as described above (*n* = 3). The peak area of the analytes on the back tube should be less than 5 % of the peak area found on the first sorbent tube [17].

### Applicability

**Fig. 1:**
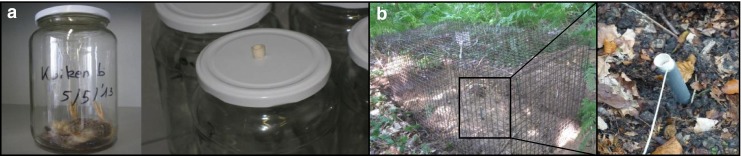
Setup of the experiment: **a** laboratory environment and **b** field experiment

Different animal species (rabbits, mice, frogs, chicks, robins, and a sturgeon) were decomposed in a glass jar in laboratory environment (Fig. 1a). The air above the animals was sampled twice a week during the first month. Afterwards, the air was sampled once a week until the third month and afterwards, once a month until no new VOCs were seen. This was performed by drawning 2 L of air through the sorbent tube (20 min at 100 mL/min). Because of its size, different organs from a pig and human were removed and stored in glass jars. The same sampling method applies to those remains. The compounds were identified using NIST98 mass spectral library (for correct identification, the MS spectra match factor was minimum 70 % and the retention time shift was limited to 5 %).

Outdoor experiments were performed to evaluate the impact of the environment on the VOCs originating from the decomposition process. The field experiments were conducted in woods in the province of Brabant (Belgium), a field used by the DVI. The animals (pigs, rabbit, rat, mice, frogs, woodpecker, mole, and a pigeon) were buried and covered with 10 cm of soil. Metal mesh cages were put on top of the graves to avoid disturbance by scavengers. A plastic tube was put in the soil above the animal and was closed with a septum (Fig. 1b). Through this tube, the air above the animals was sampled once a week using the ACTI-VOC pump (15 min at 100 mL/min). Additionally, a sorbent tube remained in the plastic sampling tube during the whole week to allow passive adsorption.

## Results and discussion

### Method development

SPME was first considered as a possible sampling technique. Nevertheless, TD was chosen since the adsorbing capacity of sorbent tubes is larger than those of SPME-fibers, thus leading to higher sensitivity. Moreover, it is possible to sample in an active way, which increases the concentration of the VOCs on the tube, again leading to higher sensitivity.

#### Glass jars

In the first setup, an erlenmeyer of 2 L was used. The erlenmeyer was completely closed. During the decomposition of a mouse, adipocere occurred. This meant that the environment was not optimal for decomposition, probably because of a lack of oxygen. Consequently, glass jars (1.062 L) were obtained and different closures were tested. Eventually, a normal screw cap closure equipped with a septum-sealed hole was the best option (Fig. 1a). Oxygen can enter the jar, and only a minimum of VOCs is lost.

#### Pump

If the volume pumped through the tube increased, the peak area of the VOCs increased with a maximum at 2 L. If the flow rate decreased, the peak area increased. Eventually, the best combination was obtained taking into account the sampling time; 20 min at 100 mL/min, resulting in 2 L of air drawn through the sorbent tubes.

#### Sorbent tubes

**Fig. 2:**
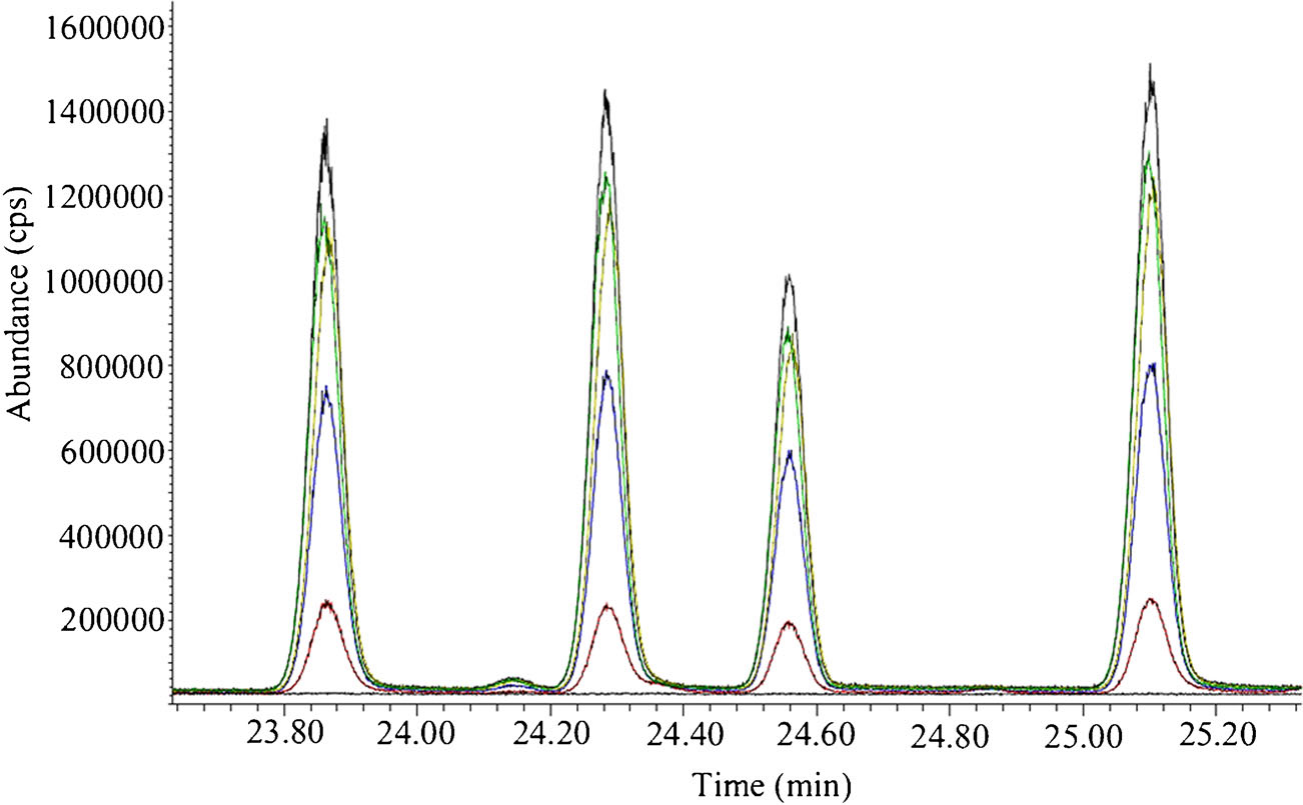
Chromatogram resulting from sorbent tubes with different types of coating: Tenax TA (*black*), Carbotrap 202 (*blue*), Carbopack B (*red*), Tenax/Unicarb (*green*), Tenax/Carboxen 1003/Carbopack B (*yellow*), and Carbosieve SIII (*dark green*)

Figure 2 shows a chromatogram that compares sorbent tubes with different types of coating material. No significant difference was observed between the peak area obtained with Tenax TA, Tenax/Unicarb, and Tenax/Carboxen 1003/Carbopack B. However, when using mice as a test model in real life samples, the chromatogram of the two latter multisorbents showed a water peak of 20 min (data not shown). Different possible solutions were examined: (1) the minimum temperature of cold trap was increased; (2) the sorbent tube was heated before sampling; (3) a cold sorbent tube without coating was put in front of the sorbent tube; (4) a sorbent tube filled with Na_2_SO_4_ was put in front of the sorbent tube; and (5) the sorbent tube was purged after the sampling. All these solutions resulted in a water peak smaller than 20 min, but still too wide (±15 min) to guarantee reliable analyte identification. Therefore, Tenax TA was chosen as the optimal sorbent tube coating.

#### GC columns

**Fig. 3:**
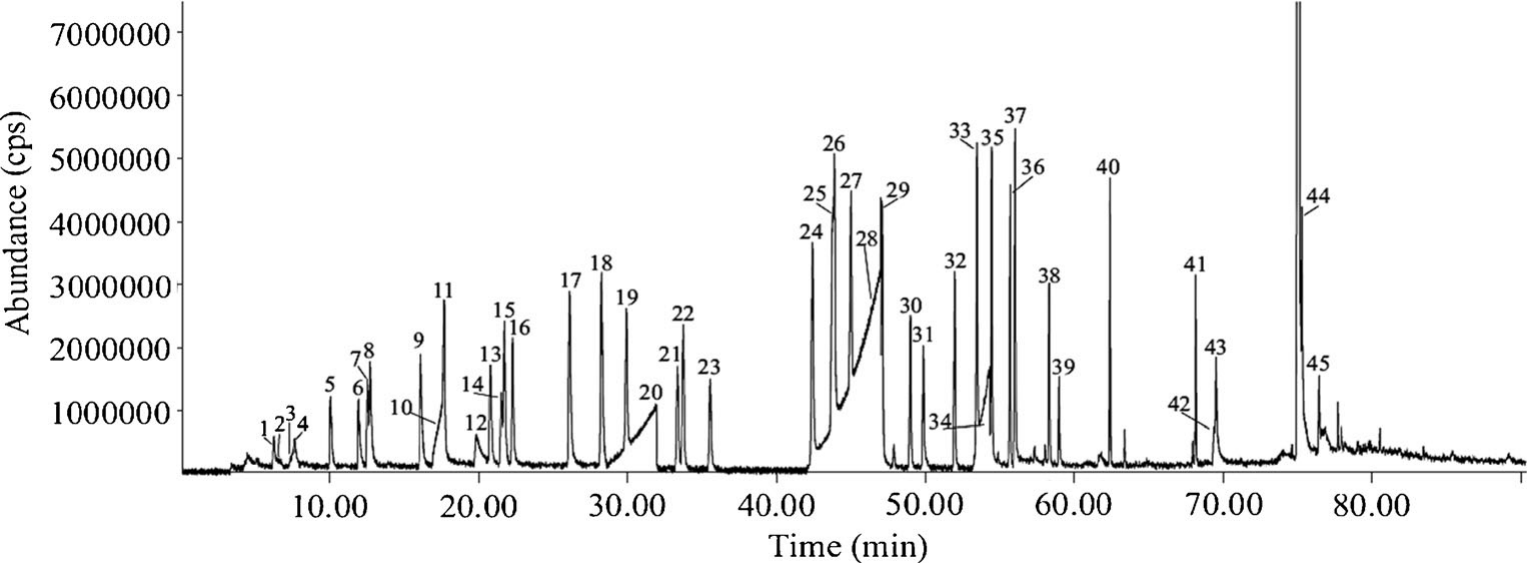
Chromatogram of method development solution. *1* Pentane, *2* ethanol, *3* dimethyl sulfide, *4* propan-2-ol, *5* hexane, *6* butanal, *7* butan-2-one, *8* ethyl acetate, *9* benzene, *10* acetic acid, *11* heptane, *12* butan-1-ol, *13* pentan-2-one, *14* pentanal, *15* ethyl propionate, *16* propyl acetate, *17* dimethyl disulfide, *18* toluene, *19* octane, *20* propanoic acid, *21* hexan-3-one, *22* ethyl butyrate, *23* hexanal, *24* ethylbenzene, *25* 1,4-dimethylbenzene, *26* 1,3-dimethylbenzene, *27* nonane, *28* butanoic acid, *29* 1,2-dimethylbenzene, *30* heptan-2-one, *31* heptanal, *32* methylpropyl disulfide, *33* propylbenzene, *34* pentanoic acid, *35* 4-ethyltoluene, *36* decane, *37* 2-ethyltoluene, *38* octan-2-one, *39* octanal, *40* undecane, *41* ethyl octanoate, *42* decan-2-ol, *43* naphthalene, *44* ethyl decanoate, and *45* biphenyl

**Fig. 4:**
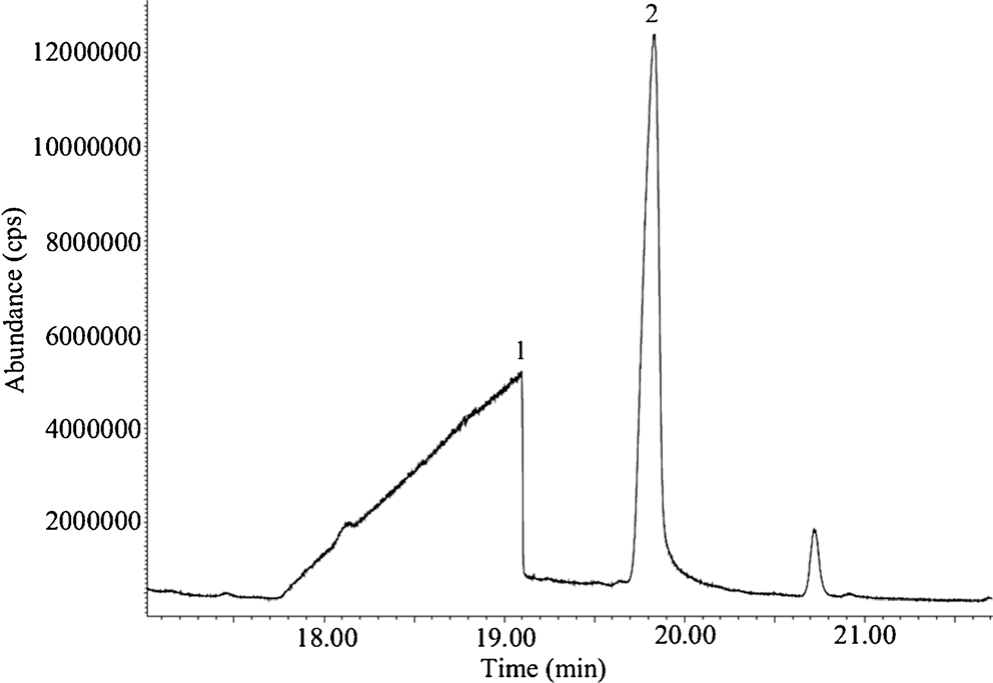
Chromatogram showing the peak difference of *1* butanoic acid and *2* trimethylsilyl butyrate

Since the TD is directly connected to the GC column via a transfer line, the pressure delivered by the TD had to be compatible with the pressure needed for the GC column, meaning that the internal diameter of the GC column had to be minimal 0.21 mm. On theVF-5ms and VF-35ms columns, the alkanes and ketones with higher molecular weights were eluted separately, but the smaller and more polar compounds eluted together with the solvent. On the other hand, all the compounds could be separated on the VF-624ms column, with exception of one co-elution (1,3- and 1,4-dimethylbenzene) (Fig. 3). This column was selected for further analyses. However, peak fronting was seen for acidic compounds. The solution for this problem will be derivatization (Fig. 4). In the future, a derivatization method will be developed to analyze the VOCs of the human and animal remains additionally to the analysis as described in this paper.

### Method validation

#### Selectivity

The analysis of the tube of a blank jar revealed the presence of a few compounds. Some analytes originated from the jar itself (1,3-diacetyloxypropan-2-yl acetate, 2-ethylhexan-1-ol, 6-methyl-5-hepten-2-one) or from the laboratory environment (acetic acid, dichloromethane, ethanol, trichloromethane), and some were degradation products of the sorbent tube itself (benzaldehyde, acetophenone, and higher aldehydes (octanal, nonanal, decanal)) [17, 18].

#### Recovery

The recovery range is 3 to 106 % for low concentrations and 6 to 95 % for high concentrations (Table 1). These lower recoveries can be explained by the volatility of the compounds and their interaction with the adsorbent. Butanoic acid and octanal have the highest boiling points and the lowest vapor pressures, resulting in a low volatility (Table 2). The interaction with the adsorbent depends on the lipophilicity of the compounds; butanoic acid has also a low log *P* (Table 2). The combination of these properties can explain the low recoveries. A solution to enhance these recoveries is the addition of a derivatization step, which will be investigated in the future.

**Table 1:** Summary of method validation data for low (L) and high (H) concentrations

VOC	Percentage of recovery		Time (days) stability		%RSD		%RSD		*R* ^2^	LOD (ppb)	LOQ (ppb)
					Rep		Int prec				
	L	H	L	H	L	H	L	H			
Heptane	105	73	3	4	14	8	21	19	0.976	0.02	0.075
Butan-1-ol	57	89	2	3	15	12	13	25	0.897	0.13	0.25
Pentanal	62	87	2	1	11	12	19	20	0.928	0.006	0.075
Pentan-2-one	69	83	4	3	13	10	21	21	0.937	0.02	0.075
Ethyl propionate	73	82	4	3	13	11	16	22	0.919	0.05	0.075
Dimethyl disulfide	44	88	4	4	14	11	21	24	0.845	0.03	0.075
Butanoic acid	3	6	1	4	24	28	47	41	–	–	–
1,2-Dimethylbenzene	89	95	4	4	15	10	20	22	0.942	0.004	0.075
Octanal	32	59	2	2	8	21	24	20	0.920	0.04	0.075

**Table 2:** Boiling point, vapor pressure, and log *P* of the analytes in the validation solution

VOC	Boiling point (°C)	Vapor pressure (mmHg)	Log *P*
Heptane	99	40	4.3
Butan-1-ol	118	4	0.8
Pentanal	103	26	1.5
Pentan-2-one	103	27	0.9
Ethyl propionate	99	20	1.2
Dimethyl disulfide	110	22	1.8
Butanoic acid	164	0.4	0.8
1,2-Dimethylbenzene	144	7	3.1
Octanal	171	2	2.8

#### Stability

The compounds were stable during 1–4 days (Table 1). The aldehydes were the least steady, but their stability was still acceptable since the VOCs stay adsorbed on the tube for maximum 24 h before the VOCs are desorbed. The instability of aldehydes is known and can be declared by oxidation in air and degradation upon storage through polymerization and acetal formation [19, 20].

#### Linearity and sensitivity

To account for unequal variances across the concentration range, a weight factor was used. In this method, the weight factor 1/y^2^ was found to be the most appropriate. The *R* ^2^ is above 0.9 for every compound except for butan-1-ol (Table 1). The LOQ was 0.075 ppb except for butan-1-ol (0.25 ppb) (Table 1). LOD values ranged from 0.004 ppb for 1,2-dimethylbenzene to 0.13 ppb for butan-1-ol (Table 1). For butanoic acid, it was not possible to make a calibration curve in underivatized analysis.

#### Repeatability and intermediate precision

Most of the VOCs showed a repeatability and precision lower than 25 % (Table 1). This is in agreement with the EPA TO-17 guidelines [17]. Butanoic acid is the exception because, as described in “Method development,” the peak has not a Gaussian shape.

#### Safe sampling volume

A breakthrough value less than 5 % is recommended [17]. When 2 L of air was drawn into the sorbent tube, the standard solution was not visible in the back tube. Hence, the sampling volume is beneath the SSV for those compounds.

### Applicability

#### In laboratory environment

A different number of decomposition compounds were identified for each species. A combination of multiple chemical classes was found for all decomposing animal remains (i.e., alkanes, alkenes, aromatic compounds, cyclic compounds, aldehydes, ketones, alcohols, S-containing compounds, N-containing compounds, acids, and esters). In our study of human remains, which is still ongoing, already 135 VOCs released by human remains were identified during the first 52 days of decomposition. Compared to most studies where the VOCs of human remains were identified, the compounds were similar to those found in our study [8–16].

**Fig. 5:**
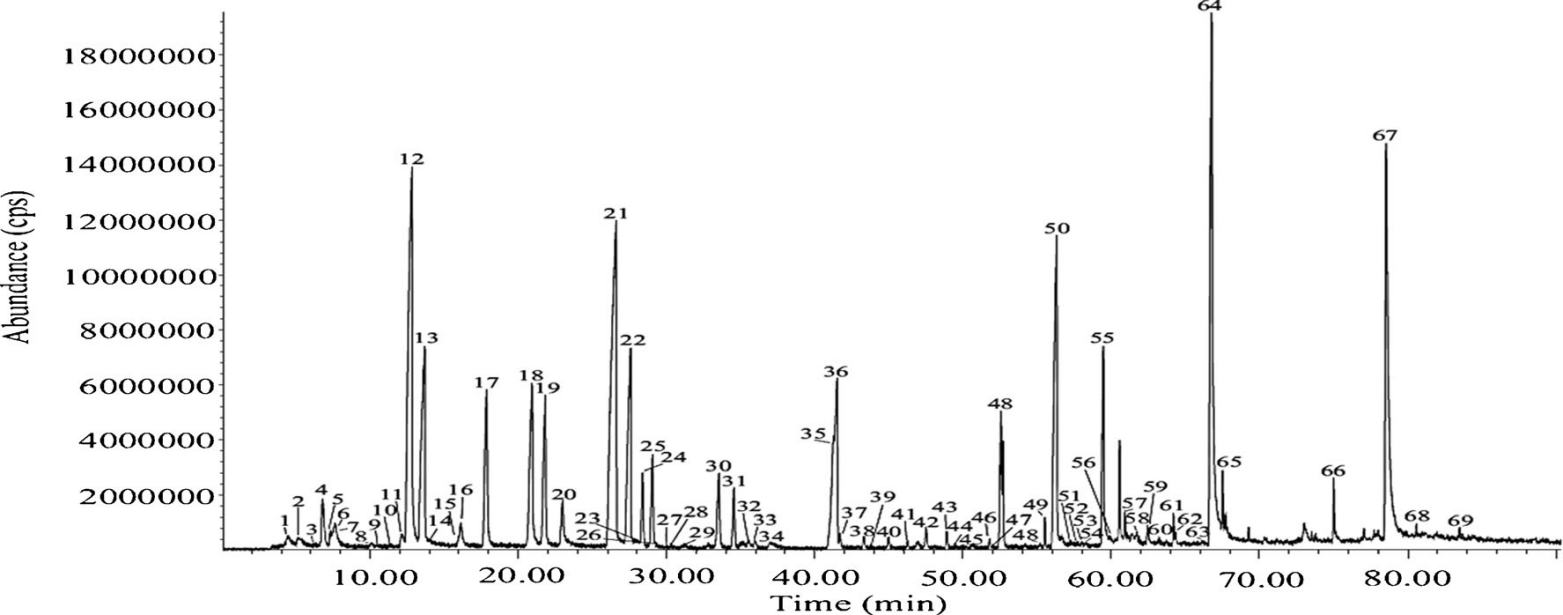
Chromatogram of VOCs encountered after 115 days of decomposition of a sturgeon. *1* Water, *2* methanethiol, *3* pentane, *4* 1,3-pentadiene, *5* propanone, *6* propan-2-ol, *7* dimethyl sulfide, *8* hexane, *9* thiirane, *10* 3-methylfuraan, *11* 2-methylfuraan, *12* butan-2-one, *13* butan-2-ol, *14* chloroform, *15* 2-methylpropan-1-ol, *16* benzene, *17* 3-methylbutan-2-one, *18* pentan-2-one, *19* pentan-3-one, *20* pentan-2-ol, *21* dimethyl disulfide, *22* 4-methylpentan-2-one, *23* 3-methyl-3-buten-1-ol, *24* methylbenzene, *25* 3-methyl-pentan-2-one, *26* 2-methylthiophene, *27* octane, *28* 3-methylthiophene, *29* 4-methylpentan-2-ol, *30 S*-methylthiopropanethioate, *31* hexan-2-one, *32* hexanal, *33* cyclopentanone, *34* hexan-2-ol, *35* 5-methylhexan-3-one, *36 S*-methyl-2-methylpropanethioate, *37* 4-methylhexan-3-one, *38* 2-methylcyclopentanone, *39* ethylbenzene, *40* 5-methylhexan-2-one, *41* heptan-4-one, *42* 2,4-dithiapentane, *43* heptan-2-one, *44* 1-methylthiopentane, *45* heptan-2-ol, *46* 2-methylheptan-4-one, *47* methylisopropyl disulfide, *48 S*-methyl-3-methylbutanethioate, *49* 6-methylheptan-2-one, *50* dimethyl trisulfide, *51* benzaldehyde, *52* octan-3-one, *53* 6-methyl-5-hepten-2-one, *54* octan-2-one, *55* phenylamine, *56* benzonitrile, *57* cycloheptanone, *58* 2-ethylhexan-1-ol, *59* phenol, *60* tetramethylpyrazine, *61* 1-phenylethanone, *62* nonan-2-one, *63* methylpentyl disulfide, *64* benzylalcohol, *65* 1-phenylpropan-2-one, *66* 1,3-diacetyloxypropan-2-yl acetate, *67* 3-methylindole, *68* hexadecane, and *69* heptadecane

**Fig. 6:**
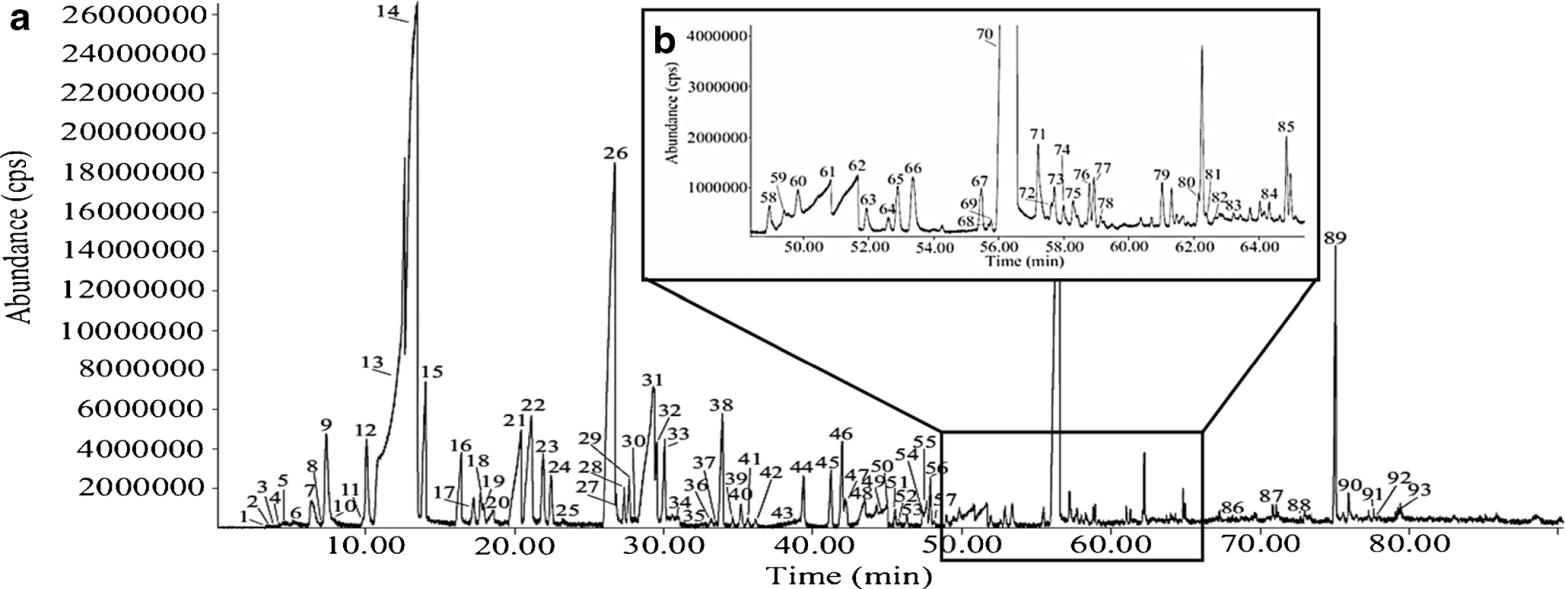
Chromatogram of VOCs encountered after 52 days of decomposition of human remains. *1* Oxygen and nitrogen, *2* carbon dioxide, *3* hydrogen sulfide, *4* sulphur dioxide, *5* water, *6* ethanal, *7* ethanol, *8* propanal, *9* propanone, *10* carbon disulfide, *11* 2-methylpropanal, *12* hexane, *13* propan-1-ol, *14* butan-2-one, *15* butan-2-ol, *16* 2-methylpropan-1-ol, *17* 3-methylbutanal, *18* heptane, *19* 3-methylbutan-2-one, *20* acetic acid, *21* butan-1-ol, *22 S*-methylethanethioate, *23* ethyl propionate, *24* propyl acetate, *25* pentan-2-ol, *26* dimethyl disulfide, *27* 3-hydroxybutan-2-one, *28* 4-methylpentan-2-one, *29* ethyl-2-methylpropionate, *30* pyridine, *31* 3-methylbutan-1-ol, *32* 2-methylbutan-1-ol, *33* octane, *34* propanoic acid, *35* 2-octene, *36* pentan-1-ol, *37 S*-methylpropanethioate, *38* ethyl butyrate, *39* hexan-2-one, *40* propyl propionate, *41* hexanal, *42* butyl acetate, *43* 2-methylpropionic adid, *44* methylethyl disulfide, *45* ethyl-2-methyl butyrate, *46* ethyl-3-methyl butyrate, *47* butane-2,3-diol, *48* butane-1,3-diol, *49* butanoic acid, *50* nonane, *51* 3-methylbutan-1-ol, *52* 2-methylbutan-1-ol, *53* heptan-4-one, *54* methylisopropyl disulfide, *55* 2,4-dithiapentane, *56* propyl butyrate, *57* ethyl pentanoate, *58* heptan-2-one, *59* 1-methylthiopentane, *60* heptanal, *61* 3-methylbutanoic acid, *62* 2-methylbutanoic acid, *63* methylpropyl disulfide, *64 S*-methyl-3-methylbutanethioate, *65* propyl-2-methylbutyrate, *66* propyl pentanoate, *67* 1-decene, *68* 6-methylheptan-2-one, *69* decane, *70* dimethyl trisulfide, *71* heptan-1-ol, *72* 1-octen-3-ol, *73* methyl 2-butyl disulfide, *74* 6-methyl-5-hepten-2-one, *75* octan-2-one, *76* 1-methylthiohexane, *77* 1-methyl-4-(1-methylethenyl)-cyclohexene, *78* heptane nitrile, *79* 2-ethylbutan-1-ol, *80* 1-undecene, *81* undecane, *82* phenol, *83* tetramethylpyrazine, *84* nonan-2-one, *85* 1-methylthioheptane, *86* ethyl octanoate, *87* dimethyl tetrasulfide, *88* undecan-2-one, *89* 1,3-diacetyloxypropan-2-yl acetate, *90* indole, *91* 1-pentadecene, *92* pentadecane, and *93* tridecan-2-one

In contrast with the studies of Vass et al., Stratheropoulos et al., and Hoffman et al., cyclic hydrocarbons, halogenetic, and nitrogen-containing compounds were not detected yet in our human remains study, notwithstanding the fact that the described method can detect these compounds since they were found in animal remains [8–12, 14]. Thus, it can be hypothesized that cyclic and nitrogen-containing compounds might still be released after the studied 52 days. Hoffman et al. and Degreeff et al., who used SPME as sampling method, detected no noncyclic hydrocarbons [14, 15]. Comparing to Vass et al. and Stratheropoulos et al., where non-branched and branched alkanes were found, only non-branched were detected in our study of human remains, whereas in animal remains, also methyl-branched alkanes were found [8–12]. Comparable alcohols and esters were found in our study and the study of Stratheropoulos et al. [10–12]. In contrast with Cablk et al., methyl-branched alcohols were identified in the human remains [16]. In our study, also butan-1,3-diol and butan-1,2-diol were detected. Interestingly, those compounds were not published in another study. Furthermore, in our study, a larger number of ketones were identified in comparing with previously published studies, except for Cablk et al., who also found ketones with higher chain lengths (up to undecan-2-one). However, Cablk et al. did not identify ketones lower than heptan-2-one [16]. Remarkably, 3-hydroxybutan-2-one was not published in any study but was detected in our study. In comparison with the studies where human remains were investigated, a larger number of sulfides were identified in our study. Also −thioles and methylthioalkanes were well represented in the S-containing compound group of our study. In Figs. 5 and 6, a represantative chromatogram of the VOCs of respectively a sturgeon (decomposing for 115 days) and human remains (decomposing for 52 days) is shown.

#### Field experiments

In the samples of the cadavers buried outdoors, the concentrations were, as expected, lower than in the glass jars (range of 0.15 to 5 ppb in laboratory environment and range of >0.075 to 2.5 ppb outdoors). This can be explained by the air volume which is minimized in the glass jars. Conversely, outside the laboratory, the air volume is not limited; the compounds need to diffuse through the soil, which takes more time, and the compounds can diffuse to other directions than upward.

Via passive sampling (when the sorbent tube remained in the plastic tube above the cadaver for 1 week), more compounds can be detected than during active sampling using the pump (15 min, 100 mL/min). This is probably because the VOCs are not concentrated in a closed environment.

## Conclusions

The presented method, using TD-GC/MS, is a validated and repeatable method that can be applied for a wide spectrum of compounds and therefore to identify the variety of VOCs that are released during the decomposition of human and animal remains. The sensitivity for most compounds is around 0.075 ppb. Because the VOCs found in laboratory environments are in the range of 0.15 to 5 ppb, the method is sensitive enough. This method is already used in laboratory environment to analyze the VOCs released from animals. More research is needed to identify (a) marker(s) specific for human decomposition and to investigate the impact of the environment on the decomposition process and the VOCs that are released.
